# Bioimpedance analysis for identifying new indicators of exercise-induced muscle damage

**DOI:** 10.1038/s41598-024-66089-8

**Published:** 2024-07-03

**Authors:** Shota Yamaguchi, Takayuki Inami, Hiroyuki Ishida, Naoya Nagata, Mitsuyoshi Murayama, Akihisa Morito, Satoshi Yamada, Naohiko Kohtake

**Affiliations:** 1 Institute of Physical Education, Keio University, Yokohama, Japan; 2 Sports Medicine Research Center, Keio University, Yokohama, Japan; 3 Taisho Pharmaceutical Co., Ltd, Tokyo, Japan; 4 Graduate School of System Design and Management, Keio University, Yokohama, Japan

**Keywords:** Eccentric exercise, Exercise-induced muscle damage, Bioimpedance analysis, Reactance, Biomarker, Correlation analysis, Physiology, Biomedical engineering, Electrical and electronic engineering

## Abstract

A noninvasive, immediate, and convenient method for assessing muscle tissue status during exercise-induced muscle damage (EIMD) has not been established. This study was designed to assess and determine parameters suitable for measuring EIMD after eccentric exercise, using multi-frequency bioimpedance analysis (BIA). Thirty-five young male participants performed dumbbell exercises with their left arm, and their BIA parameters were measured at various time points up to 168 h post exercise using a multi-frequency BIA device. At all-time points, intra and extracellular water content was greater in the left arm than in the right arm, whereas the impedance, reactance, resistance, and phase angle were lower in the left arm than in the right arm. Established EIMD indices, such as maximal isometric voluntary contraction, were measured and used in correlational analyses. Only reactance was correlated with biomarkers, indicating muscle damage (r =  − 0.56 to − 0.49). Furthermore, reactance was found to correlate well with indirect indicators of EIMD, suggesting that it may be a suitable marker for evaluating EIMD. However, the relationship with the limited evaluation indices employed in this study is constrained. Future studies should investigate the correlation between reactance and direct damage indicators, such as structural damage, observed in biopsies.

## Introduction

Forceful eccentric contractions induce inflammation and damage in myofibrils, leading to exercise-induced muscle damage (EIMD), which causes pain, loss of strength, and reduced range of motion (ROM) at onset^1^. Detailed observation of the damage at the cellular level requires a muscle biopsy; however, this is highly invasive, and the measurement environment is limited^2^. Therefore, indirect muscle damage markers, such as levels of creatine kinase^3^, myoglobin^4^, and urinary titin N-terminal fragment (UTF)^5,6^, have been used as indicators of muscle cell membrane damage. Nonetheless, these biomarkers are difficult to discriminate from those indicating localized damage and are not suitable for immediate feedback because measurement results are obtained after several days^6^.

Bioimpedance analysis (BIA) estimates body composition by applying a small electric current to the body and measuring electrical characteristics, such as reactance, resistance, and impedance. Reactance, an important component in BIA, reflects the electrical properties derived from the capacitance of the cell membrane and is useful for assessing cell mass, health, and nutritional status^7^. Cell membranes act as capacitors in electrical circuits in vivo, producing phase shifts between current and voltage. This is called the phase angle (PhA) and refers to the angle of impedance exhibited by biological tissue to alternating current, reflecting the ability of the current to pass through the cell membrane and the distribution state of ions inside and outside the cell. Several studies have established that measuring PhA is a valid noninvasive method for quantifying cell health^8,9^ and evaluating the grade of muscle injury, as its value decreases when the integrity of the myocyte membrane is compromised^9,10^. Recently, evaluation of localized parts of the body was attempted^11^, which may overcome some of the problems posed by using biomarkers alone in the evaluation of EIMD.

Only few previous studies have evaluated the biological changes caused by EIMD using BIA^12^. Extracellular water (ECW) content increases at the onset of EIMD, and there is a positive correlation (r = 0.654) between peak ECW content and peak creatine kinase^11^. While examining the process of recovery from EIMD, it is important to consider physiological changes; however, to date, no study has used BIA in combination with markers, such as muscle strength markers, to assess recovery from EIMD. Yamada et al.^13^ found that, among PhA, resistance, and reactance, reactance had the strongest correlation with maximum muscle power in participants aged 20–70 years old (r = 0.881–0.898). Reactance quantifies the resistance of cell membranes to alternating current across a spectrum of frequencies, serving as an indicator of muscle cell health and integrity. Intact cell membranes in healthy muscle cells effectively maintain electrochemical gradients crucial for muscle contraction, which depend on rapid shifts in membrane potential. Consequently, elevated reactance values correlate with enhanced muscle cell quantity and quality, thereby augmenting muscle power output. Low frequencies do not penetrate the cell membrane, whereas high frequencies do; therefore, a detailed analysis of frequency characteristics can discriminate between intra- and extracellular changes^14,15^. Although there is a lack of detailed knowledge on the relationship between the various indices of EIMD, such as muscle strength, and multiple BIA parameters and frequency characteristics, the above findings suggest that changes in reactance at low frequencies could indicate substantial EIMD. Consequently, tracking the BIA-parameters following EIMD may enhance our understanding of the electrophysiological mechanisms underlying EIMD.

Therefore, this study aimed to analyze and track BIA parameters in various frequency bands and determine the variables suitable for measuring EIMD. We hypothesized that reactance obtained at low frequencies would be most associated with changes in muscle strength and biochemical markers produced by EIMD, based on the report by Yamada et al.^13^. In this study, we used a standing multi-frequency bioimpedance meter, which is widely used in clinical settings and is highly correlated with dual-energy X-ray absorptiometry values^16^, to examine the changes in parameters in response to EIMD. If the degree of muscle damage can be predicted by BIA, it may be possible to indirectly identify the degree of damage without placing a burden on the individual during daily body composition measurements in the future.

## Results

### EIMD indices

**Figure 1:**
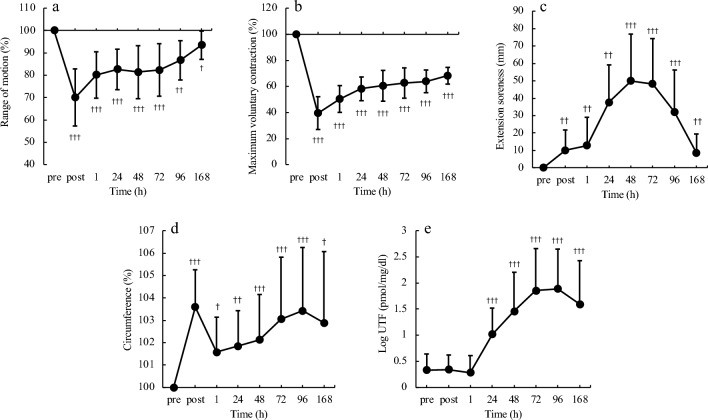
Time course of EIMD evaluating indices. (**a**) Range of motion, (**b**) maximum voluntary contraction, (**c**) soreness during elbow extension, (**d**) circumference of the upper arm, e: urinary titin N-terminal fragment, ^†^p < 0.05, ^††^p < 0.01, ^†††^p < 0.001.

The comparative results of the EIMD assessment indices are presented in Fig. 1. ROM and maximum voluntary contraction (MVC) showed the greatest decreases immediately after exercise, followed by a trend toward recovery at 24 h. Muscle soreness increased significantly immediately after exercise and reached a maximum after 48 h. Circumference peaked immediately after exercise, falling at 1 h, followed by an increase over the measured time points until a recovery trend at 168 h. The UTF showed a notable increase at 24 h, reaching a maximum at 96 h, followed by a recovery trend at 168 h.

### Cell water content

**Figure 2:**
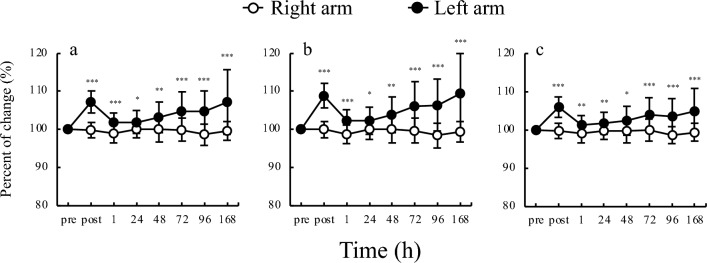
Time course TBW, ECW, and ICW. (**a**) Total body water; (**b**) extracellular water; (**c**) intracellular water; *p < 0.05, **p < 0.01, ***p < 0.001.

**Figure 3:**
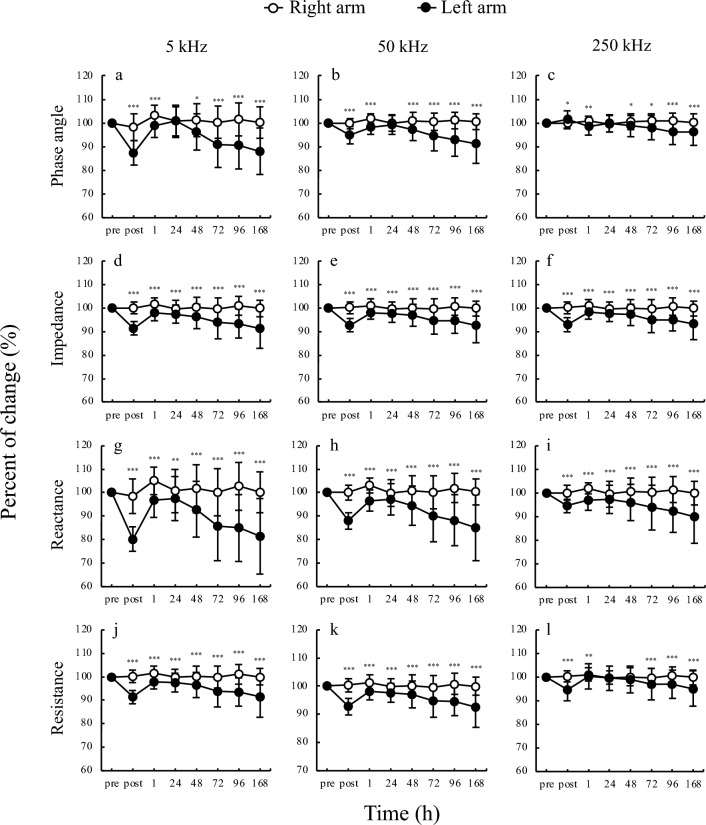
Time course of each bioimpedance analysis parameter. (**a**) 5 kHz phase angle, (**b**) 50 kHz phase angle, (**c**) 250 kHz phase angle, (**d**) 5 kHz impedance, (**e**) 50 kHz impedance, (**f**) 250 kHz impedance, (**g**) 5 kHz reactance, (**h**) 50 kHz reactance, (**i**) 250 kHz reactance, (**j**) 5 kHz resistance, (**k**) 50 kHz resistance, (**l**) 250 kHz resistance, * p < 0.05, ** p < 0.01, *** p < 0.001.

The results of the comparison between right arm and left arm for cell water content are shown in Fig. 2. The total body water (TBW), ECW, and intracellular water (ICW) for left arm was higher than that for right arm at all time points. The comparison between right arm and left arm for BIA values measured over time are shown in Fig. 3. PhA for left arm was lower than that for right arm at all frequencies at each time point, except at 24 h. Impedance and reactance for left arm were lower than that for right arm at all frequencies and time points. The left arm had lower resistance than right arm at all time points at 5 kHz and 50 kHz; at 250 kHz, left arm had lower resistance than the right arm immediately post exercise, and at 1, 72, 96, and 168 h post exercise.

### Comparison of ECW and ICW relative to TBW

**Figure 4:**
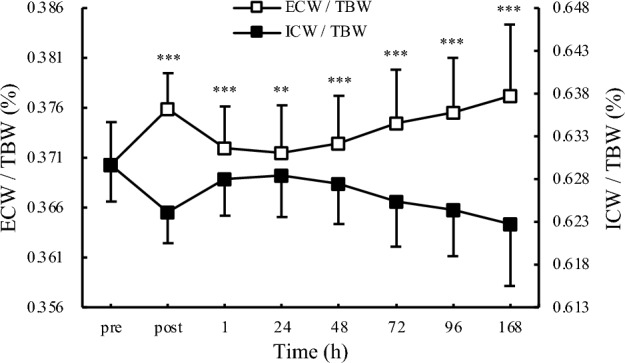
Ratio of extra- and intracellular water to total body water. *TBW* total body water, *ECW* extracellular water, *ICW* intracellular water, *p < 0.05, ** p < 0.01, *** p < 0.001.

The comparison results between ECW/TBW and CW/TBW are shown in Fig. 4. ECW/TBW exceeded ICW/TBW at all time points, except pre-test.

### Bioelectrical impedance parameters at each frequency and EIMD index

**Figure 5:**
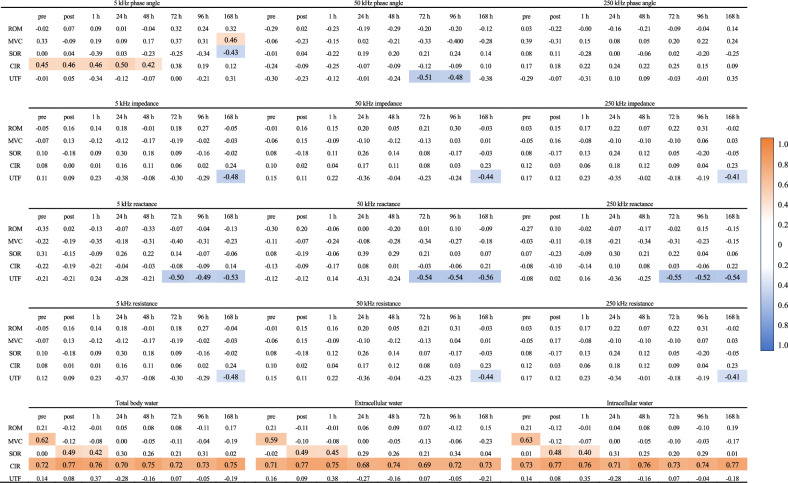
Relationship between the electrical characteristic parameters and EIMD evaluation indices. Areas with higher levels of correlation coefficient are depicted as darker, and those with lower levels of correlation coefficient as lighter. Areas with no statistically significant differences are not colored. *ROM* range of motion, *MVC* maximum voluntary contraction, *SOR* muscle soreness, *CIR* upper arm circumference, *UTF* urinary titin N-terminal fragment. *p < 0.05, **p < 0.01.

Figure 5 presents the results of the correlation analysis of the relationship between the electrical characteristic parameters and the EIMD evaluation indices. Due to the large number of correlations identified, only parameters with three or more correlations are reported. Positive correlations were found between PhA 5 kHz and circumference (r = 0.421–0.495). Negative correlations were found between reactance at 5 kHz and UTF (r =  − 0.532 to 0.493), reactance at 50 kHz and UTF (r =  − 0.559 to − 0.543), and reactance at 250 kHz and UTF (r =  − 0.547 to − 0.519). A positive correlation was found between TBW and circumference (r = 0.701–0.766), ECW and circumference (r = 0.677–0.766), and ICW and circumference (r = 0.725–0.765).

## Discussion

The purpose of this study was to follow the temporal changes in BIA parameters obtained at different frequencies with the onset of EIMD and to determine BIA parameters suitable for the assessment of EIMD. We hypothesized that the reactance obtained in the low-frequency band (5 kHz in this study), which is less likely to penetrate muscle cell membranes, would have the strongest relationship with the measures used to assess EIMD. Although our results showed high correlations between UTF with both reactance and PhA, no clear differences were observed in the ability of the three frequencies (5 kHz, 50 kHz, and 250 kHz) to detect EIMD.

In the present study, participants were seated on an arm curl bench as described by Nosaka et al.^17^, and an exercise task was performed in which the examiner lifted the dumbbells during flexion, and the participant controlled and lowered the dumbbells for 5 s during extension. The results showed that ROM was most restricted immediately after the eccentric exercise (Fig. 1a), muscle soreness peaked 48 h after the eccentric exercise (Fig. 1c), and UTF peaked 96 h after the eccentric exercise (Fig. 1e). These temporal changes were similar to those reported in previous studies^5,18^ at the onset of EIMD. In addition, MVC decreased by 61.5% immediately after the exercise task and did not recover to baseline, even at 168 h (Fig. 1b). Damas et al.^19^ performed a cluster analysis based on the rate of decrease in MVC during the onset of EIMD and identified low, intermediate, and high responder groups. They reported that the high responder group had a 57.8% decrease in MVC. This suggests that EIMD was more severe in the present study than in the high responder group in the previous study. Eccentric contractions result in primary mechanical and secondary metabolic damages as a consequence of the primary damage. When damage occurs, functional measures such as muscle strength and joint ROM are significantly reduced immediately after exercise and do not recover until 2–7 days post-exercise. Conversely, no muscle damage occurs in concentric contractions, and the decline is only temporary due to fatigue immediately after exercise^20^. Given the similarity of the present results to the post-injury temporal changes shown in previous studies, the observed effects were inferred to be predominantly influenced by damage rather than fatigue.

In this study, TBW, ECW, and ICW, all increased considerably immediately after exercise in the left arm and decreased 1 h later. However, these indices increased over time (Fig. 2). The changes observed in this study may also be attributed to vasodilation and increased permeability because they generally occur under the influence of multiple substrates (such as histamine and kinin) and induce swelling at the site of injury^21^. Furthermore, analysis of the balance between ICW and ECW content from our results showed that ECW increased more than ICW after eccentric exercise (Fig. 4). A study by Shiose et al.^11^, which reported changes in TBW, ECW, and ICW during EIMD, also reported an increase in ECW/TBW without a decrease in ICW, supporting our results. A previous study^22^ has shown that muscle mass is reduced when ICW decreases and ECW/TBW increases simultaneously; such changes are often observed in cases of muscle atrophy and sarcopenia in older individuals. In contrast, an increase in ECW/TBW without a decrease in ICW is thought to indicate an increase in intracellular and extracellular water content combined due to vasodilation and increased permeability associated with acute or chronic inflammation^23^. In this study, an increase in ECW and TBW occurred without a decrease in ICW, suggesting that an acute inflammatory response occurred immediately after eccentric exercise, and a chronic response occurred after 24 h.

In the present study, all BIA parameters showed similar patterns of change over time, although the magnitudes of change were different (Figs. 2, 3). The impedance, resistance, reactance, and PhA values obtained by applying a weak current in vivo provides information on biological functions^24^, and the results of this study confirm that each BIA parameter, which changed substantially during the onset of EIMD, can provide a large amount of detail on cell processes. For instance, reactance serves as an indicator of cell membrane capacitance, which in turn reflects the structural integrity and functional capacity of the cell membrane. Conversely, resistance is associated with the volume of ECW and the overall hydration state of the tissues. The PhA and impedance collectively provide a comprehensive measure of both reactance and resistance, thus furnishing insights into the health and integrity of cellular structures. As a computational parameter, PhA demonstrates greater sensitivity to variations in reactance, whereas impedance is more responsive to changes in resistance. In the context of this study, reactance was observed to diminish following the onset of EIMD owing to compromised cell membranes, and resistance likewise declined as a consequence of edema formation. Consequently, we hypothesized that both PhA and impedance decreased in response to these physiological alterations.

This study was based on the hypothesis that there would be differences in the correlations of BIA parameters with EIMD depending on the frequency, and that the BIA parameters, especially those in the low-frequency band, would be strongly correlated with the EIMD assessment index. However, the correlations shown in Fig. 5 do not support this hypothesis. Since the low-frequency component does not penetrate the muscle cell membrane, in stark contrast to the high-frequency component, it is understood that frequency characteristics can be used to differentiate between intra- and extracellular changes^24^. Therefore, it was inferred that the low-frequency component would have a higher resistance in cells with high integrity and a lower resistance in cells in a highly damaged state, which would also affect the detection power. However, the results of the present study did not reveal such characteristics. In contrast, bioelectrical impedance spectroscopy (BIS) employing 256 frequencies provides detailed data, including the electrical properties index, characteristic frequency, and membrane capacitance^25^, which cannot be obtained using conventional BIA. The electrical properties index, in particular, allows for a nuanced evaluation at the cellular level by directly reflecting the health and capacitance characteristics of cell membranes. Although frequency variations are less critical, indices such as the electrical properties index, which more precisely assess cellular integrity, may enable a more detailed analysis of muscle damage.

Although PhA has been tested in sports medicine to assess muscle strain^9^, to the best of our knowledge, this is the first time that BIA parameters have been validated for characterizing EIMD. All BIA parameters showed noticeable changes after EIMD, with reactance having the strongest relationship with UTF. Reactance represents the resistance to current caused by the phospholipid bilayer structure of the myocyte membrane; the higher the reactance, the lower the current-carrying capacity^24^. Sarcolemma disruption has been observed during the onset of EIMD^26^. Eccentric exercise causes mechanical strain on muscle fibers and overloads the sarcolemma and T tubules by overstretching the muscle segments. This causes opening of stretch-activated channels and disruption of membranes, allowing Ca^2+^ to enter the cytoplasm and activate calpain enzymes. These enzymes degrade contractile proteins and cause a long-term loss of muscle strength^27^. Therefore, in this study, the decrease in reactance observed at the onset of EIMD may have been caused by the sarcolemma disruption. However, although a theoretical correlation is hypothesized, in practice, the time course of UTF leakage and BIA changes differ significantly. Therefore, while there are similarities, they do not completely coincide, and BIA merely estimates the damage to the cell membrane. Further investigation is needed in this regard.

While this study has provided valuable insights into the application of BIA for monitoring EIMD, it is important to acknowledge its limitations. First, the use of a single exercise intensity (50% of maximal voluntary contraction) and a fixed protocol of 5 sets of 10 repetitions may not fully represent the range of responses at higher exercise intensities or different training volumes. Future research should explore the effects of varying the load and adjusting the number of repetitions and sets to elucidate the dynamics of muscle damage and recovery. Second, the participants were limited to healthy young men, making the differences in performance by age indeterminate. In particular, the baseline of each BIA parameter was similar to that indicated by muscle damage status because older adults have lower cellular integrity than younger adults (as a typical example, older adults have lower PhA values than younger adults^13^). Therefore, it may be difficult to determine whether the changes in BIA parameters are due to EIMD. In future, it is necessary to broaden the age range and investigate the relationship between age and the detectability of EIMD. Third, the use of standing multi-frequency BIA as an evaluation method made it impossible to clarify the effect of frequency differences on the detectability of EIMD. As mentioned above, this limitation can be overcome by using BIS with more applicable frequencies; therefore, a detailed study, including congruency with the BIS, is needed. Fourth, while BIA and UTF both aim to quantify cell membrane damage, they measure fundamentally different aspects of this damage. BIA provides real-time information about the electrical properties of cell membranes, reflecting ongoing cellular recovery and adaptation processes. In contrast, UTF measures the degradation and leakage of titin into the urine, a process that includes additional biochemical pathways and can introduce delays and variations in the observed time course. Consequently, the fluctuations in bioimpedance parameters observed up to 168 h post-exercise likely represent ongoing recovery processes not captured by UTF, which tends to stabilize earlier. Finally, the point at which the BIA parameters completely returned to the baseline could not be determined. In the present study, BIA parameters were monitored for up to 168 h after eccentric exercise. To the best of our knowledge, no study has followed BIA parameter recovery after EIMD. BIA parameters that also indicate the inflammatory response show that recovery may take up to one month, as reported by Nosaka et al.^28^. Therefore, future follow-up studies of up to one month are needed to elucidate the timing of recovery to baseline.

In conclusion, we tested the validity of EIMD measurement indices for several BIA parameters. Among them, reactance was found to correlate well with indirect indicators of EIMD, suggesting that it may be a suitable marker for evaluating EIMD. Physicians and athletic trainers may find BIA useful for obtaining a reliable and simple assessment index for athletes. However, the relationship with the limited evaluation indices employed in this study is constrained. Future validation using BIS is needed to further increase detectability of EIMD, and further validation across a wider age range is needed to account for age-related effects.

## Methods

### Participants

Thirty-five healthy male participants (aged 23.0 ± 3.1 years; weighing 64.3 ± 8.6 kg) were enrolled in this study. We excluded participants who habitually exercised and/or trained, those with arm injuries, those with pacemakers implanted in their bodies, and those under 18 and over 35 years of age. The study was approved by the Human Ethics Committees of Keio University (21–003) and conducted in accordance with the principles of the Declaration of Helsinki. Participants were informed of the nature, aims, and risks associated with the experimental procedures before providing written informed consent.

### Experimental design

The design of measurement parameters, excluding those of BIA, used in this study were based on that of a previous study^18^ on EIMD. The study was conducted over nine days. Before the experiment, the participants took part in a familiarization session, which included muscle strength measurements. On the first day before the exercise session, a warm-up was conducted, and an eccentric exercise routine was performed involving the elbow flexor muscles. The values measured were MVC, ROM, muscle soreness, circumference, bioimpedance, and UTF. Immediately before and after the eccentric exercise routine, all parameters were measured, and the measurements were repeated at 1, 24, 48, 72, 96, and 168 h. In this study, all participants performed exercises with the left arm. For measurements immediately before and after exercise, and from days 2–8, only the left arm was measured for MVC, ROM, muscle soreness, and circumference to establish the onset of EIMD. BIA was performed on both arms to compare the differences between the right arm and left arm. The test–retest reliability of the indirect markers was calculated by comparing the values from the familiarization session with the pre-exercise values. This was done by determining the coefficient of variation (CV). The CVs were 2.62% for body weight, 1.49% for ROM, 7.02% for isometric strength, 0.00% for muscle soreness, 5.26% for circumference, and 37.45% for UTF.

### Eccentric exercise routine

In this study, the exercise involved the left arm. Participants sat on a preacher curl bench with the hip at 85° (0° = full hip extension) and the shoulder joint flexed at 45°. They were instructed not to move their bodies or upper arms during the exercise. They completed five sets of 10 eccentric exercises with dumbbells weighing 50% of the elbow joint MVC of the left arm, measured in the familiarization session. The elbow joint extended from 90° to 180° (180° = full extension) to the 60 beats per min rhythm of the metronome (i.e., extended 90° in 5 s). The examiner supported the participant's elbow flexion during the concentric phase to perform eccentric actions. All actions were repeated every 3 s, and a recovery period of 2 min was provided between each set.

### BIA

Participants wore light athletic clothing and were instructed to remove shoes and any plastic, metal, and easily removable jewelry from their bodies. A multi-frequency BIA device (InBody770, InBody Japan Inc., Japan) was used for BIA. The participants stepped onto the multi-frequency BIA device, held onto the handrails bilaterally, and remained on the device for 2 min. This analyzer used an alternate current of 250 mA and assessed impedance, reactance (Xc), resistance (R), TBW, and ECW. The parameters were measured at frequencies of 5, 50, and 250 kHz. The data were used to calculate PhA and ICW using the formula below.

$$PhA (^\circ ) = Arctangent\frac{Xc}{R}\times \frac{180^\circ }{\pi }$$

$$ ICW \, = \, TBW \, - \, ECW $$

The multi-frequency BIA device measured segmental impedances in the right arm, left arm, right leg, and left leg at all three frequencies.

### MVC evaluation

Maximal isometric elbow flexion strength was evaluated during 5 s isometric MVCs performed at an elbow angle of 90° using a handheld dynamometer (Mobie, SAKAI Medical Co., Ltd. Japan).Two trials were performed (if the difference between the two measurements exceeded 10%, a third measurement was taken), and the maximum value obtained was used.

### Active ROM

A semi-permanent marker was used to mark the center of the acromion, lateral epicondyle, and ulnar styloid. The elbow joint angle was photographed in a relaxed and flexed state to determine the active ROM. The angle formed between the line connecting the center of the acromion and lateral epicondyle and that connecting the lateral epicondylitis and ulnar styloid was calculated using the ImageJ software (version 1.39, Bethesda, Maryland, USA). After determining the relaxed and flexed angles, we subtracted the relaxed angle from the flexed angle to determine the ROM of the elbow joint.

### Muscle soreness

We measured muscle soreness by subjectively evaluating muscle damage^5^. A 100-mm visual analog scale was used to assess muscle soreness, with 0 indicating no pain and 100 representing extreme pain. Muscle soreness was measured when the participants actively extended their arms. Participants were instructed to hold their shoulder joints at 90° flexion and elbow joints at 180° active extension and to mark the perceived soreness on the visual analog scale.

### Circumference

Circumference of the upper arm was measured using a tape measure (Model R-280; Futaba, Japan) at 50% of the distance from the acromion to the lateral epicondyle of the humerus when letting the arm hang down by the side. The mean of two measurements was recorded.

### Titin N-terminal fragment excretion assay

Approximately 3 mL of urine was collected from each participant to measure UTF concentrations using an ELISA kit (Titin N-terminal Fragment Assay Kit, Immuno-Biological Laboratories Co. Ltd., Japan) in accordance with previous studies^29^. Samples were stored at − 20 ℃ for later analyses. Thawed urine samples were diluted 1:5–1:500 to ensure they were within the linear detection range. Diluted samples and standard solutions were added to each antibody-coated well of 96-well microplates, and the microplates were incubated at 37 ℃ for 60 min. The microplates were washed four times with the wash buffer, and labeled antibodies were added to each well; the microplates were incubated again at 37 ℃ for 30 min. Microplates were incubated with tetramethylbenzidine solution at room temperature (20–25 ℃) for 30 min after washing five times with wash buffer. As the final step of the ELISA procedure, the stop solution was added to each well. The absorbance was measured using a microplate reader at a main wavelength of 450 nm (Multiskan FC, Thermo Scientific, Japan). The UTF concentration was calculated using a linear regression model and urinary creatinine levels were estimated using an automated analyzer (Bio Majesty JCA-BM8060, JEOL, Japan). The UTF values were normalized relative to urinary creatine (each raw data point in urine/urinary creatine concentration)^29^.

### Statistical analysis

All data are expressed as means ± SD. Correlations between variables were analyzed using Pearson's product-moment correlation. Raw data were used for correlation analysis. Changes over time in ROM, MVC, muscle soreness, circumference, and UTF in the eccentric exercise condition were compared using one-way ANOVA. When the one-way ANOVA results indicated a significant difference, a Bonferroni post-hoc test was performed to compare the values at different time points. The changes after exercise were compared between the conditions (right arm vs. left arm) using two-way ANOVA with two factors (arm × time). If a significant interaction effect was found, a post-hoc test was performed to identify the time points of significant differences between the conditions using Bonferroni’s method. This test indicated a non-normal distribution of the UTF data; therefore, we applied a logarithmic transformation (log_10_) before analysis^5^. The statistical significance level was set at p < 0.05. All statistical analyses were performed using Predictive Analytics Software, version 28 for Windows (SPSS Japan Inc., Tokyo, Japan) (Supplementary Information S1).

### Supplementary Information

Supplementary Information.

## Data Availability

The datasets used and/or analysed during the current study are available from the corresponding author on reasonable request.

## References

[1] Clarkson PM, Hubal MJ (2002). Exercise-induced muscle damage in humans. Am. J. Phys. Med. Rehabil..

[2] Mackey AL, Kjaer M (2017). Connective tissue regeneration in skeletal muscle after eccentric contraction-induced injury. J. Appl. Physiol..

[3] Brancaccio P, Maffulli N, Limongelli FM (2007). Creatine kinase monitoring in sport medicine. Br. Med. Bull..

[4] Kanda K (2013). Eccentric exercise-induced delayed-onset muscle soreness and changes in markers of muscle damage and inflammation. Exerc. Immunol. Rev..

[5] Yamaguchi S, Suzuki K, Kanda K, Inami T, Okada J (2020). Changes in urinary titin N-terminal fragments as a biomarker of exercise-induced muscle damage in the repeated bout effect. J. Sci. Med. Sport..

[6] Yamaguchi S, Suzuki K, Kanda K, Okada J (2020). N-terminal fragments of titin in urine as a biomarker for eccentric exercise-induced muscle damage. J. Phys. Fit. Sports Med..

[7] Sanchez B (2017). Non-invasive assessment of muscle injury in healthy and dystrophic animals with electrical impedance myography. Muscle Nerve..

[8] Giaever I, Keese CR (1993). A morphological biosensor for mammalian cells. Nature..

[9] Nescolarde L (2013). Localized bioimpedance to assess muscle injury. Physiol. Meas..

[10] Di Vincenzo O, Marra M, Scalfi L (2019). Bioelectrical impedance phase angle in sport: A systematic review. J. Int. Soc. Sports Nutr..

[11] Shiose K, Tanabe Y, Ohnishi T, Takahashi H (2019). Effect of regional muscle damage and inflammation following eccentric exercise on electrical resistance and the body composition assessment using bioimpedance spectroscopy. J. Physiol. Sci..

[12] Inami T, Yamaguchi S, Kim HK, Murayama M (2022). Localized-bioelectrical impedance vector analysis on mechanical property changes after muscle injury and damage. J. Sports Med. Phys. Fitness..

[13] Yamada, Y. *et al*. Electrical properties assessed by bioelectrical impedance spectroscopy as biomar.kers of age-related loss of skeletal muscle quantity and quality. *J. Gerontol. A. Biol. Sci. Med. Sci*. **72**, 1180–1186 (2017). 10.1093/gerona/glw225 PMC5861891 28814064

[14] Yamada Y (2017). Developing and validating an age-independent equation using multi-frequency bioelectrical impedance analysis for estimation of appendicular skeletal muscle mass and establishing a cutoff for sarcopenia. Int. J. Environ. Res. Public Health..

[15] Moonen HPFX, Van Zanten ARH (2021). Bioelectric impedance analysis for body composition measurement and other potential clinical applications in critical illness. Curr. Opin. Crit. Care..

[16] Demura S, Sato S, Kitabayashi T (2004). Percentage of total body fat as estimated by three automatic bioelectrical impedance analyzers. J. Physiol. Anthropol. Appl. Human Sci..

[17] Nosaka K, Newton M (2002). Repeated eccentric exercise bouts do not exacerbate muscle damage and repair. J. Strength. Cond. Res..

[18] Inami T (2022). Changes in muscle shear modulus and urinary titin N-terminal fragment after eccentric exercise. J. Sports. Sci. Med..

[19] Damas F, Nosaka K, Libardi CA, Chen TC, Ugrinowitsch C (2016). Susceptibility to exercise-induced muscle damage: A cluster analysis with a large sample. Int. J. Sports. Med..

[20] Yamaguchi S (2020). Changes in urinary titin N-terminal fragment concentration after concentric and eccentric exercise. J. Sports. Sci. Med..

[21] Clarkson PM, Sayers SP (1999). Etiology of exercise-induced muscle damage. Can. J. Appl. Physiol..

[22] Campa F (2022). Effect of resistance training on bioelectrical phase angle in older adults: A systematic review with meta-analysis of randomized controlled trials. Rev. Endocr. Metab. Disord..

[23] Ge, Y. Z. *et al*. Extracellular water to total body water ratio predicts survival in cancer patients with sarcopenia: a multi-center cohort study. *Nutr. Metab. (Lond)*. **19**, 34 (2022). 10.1186/s12986-022-00667-3 PMC9077863 35525966

[24] Abasi S, Aggas JR, Garayar-Leyva GG, Walther BK, Guiseppi-Elie A (2022). Bioelectrical impedance spectroscopy for monitoring mammalian cells and tissues under different frequency domains: A review. ACS Meas. Sci. Au..

[25] Freeborn TJ, Milligan A, Esco MR (2018). Evaluation of ImpediMed SFB7 BIS device for low-impedance measurements. Measurement..

[26] Vierck J (2000). Satellite cell regulation following myotrauma caused by resistance exercise. Cell Biol. Int..

[27] Peake JM, Neubauer O, Della PA, Nosaka K (2017). Muscle damage and inflammation during recovery from exercise. J. Appl. Physiol..

[28] Nosaka K, Sakamoto K, Newton M, Sacco P (2001). How long does the protective effect on eccentric exercise-induced muscle damage last?. Med. Sci. Sports Exerc..

[29] Maruyama N (2016). Establishment of a highly sensitive sandwich ELISA for the N-terminal fragment of titin in urine. Sci. Rep..

